# Supplementary material for: Evaluating the performance of automated detection systems for long-term monitoring of delphinids in diverse marine soundscapes
Source: PLoS One. 2025 Jun 11;20(6):e0323768. doi: 10.1371/journal.pone.0323768 (PMC12157003; doi:10.1371/journal.pone.0323768)
Supplement: S1 File — (PDF) [file pone.0323768.s001.pdf]

Supplementary Material: Evaluating the performance of automated detection systems for long-term monitoring of delphinids in diverse marine soundscapes.

Contact author: Ellen White, elw1d23@soton.ac.uk

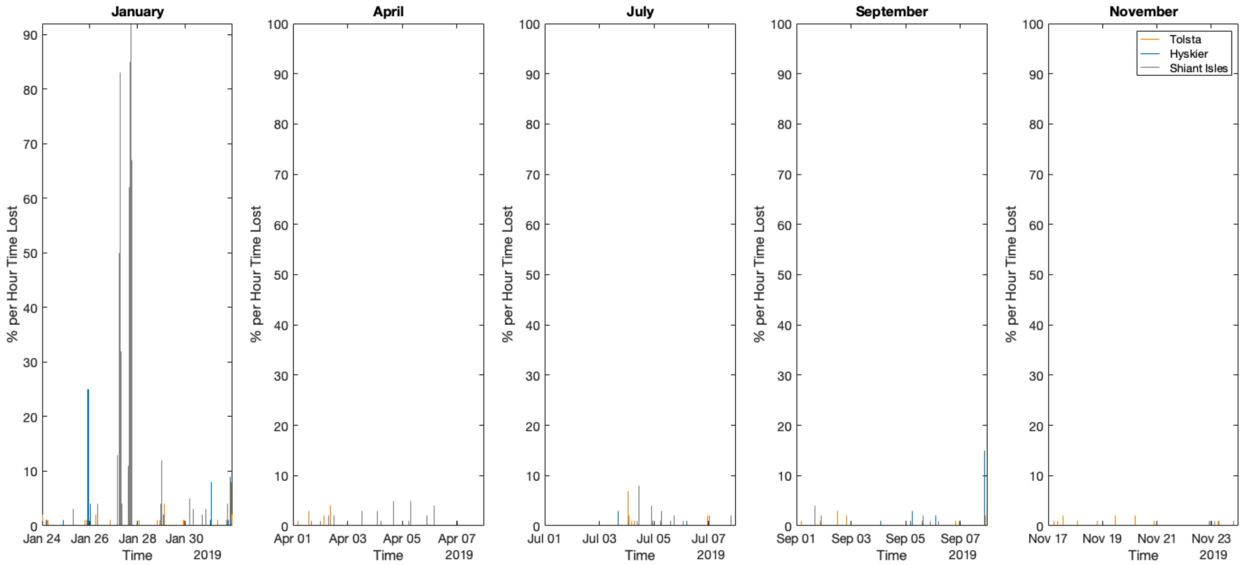

Supplementary Figure 1. The percentage of time lost on the C-POD recording platform, per season and mooring location: Tolsta, Hyskier and Shiant Isles. The C-POD does not suffer extensive time lost, with each site reporting < 10% data loss per hour between April – November. Shiant Isles reports high levels of time lost in January 2019.

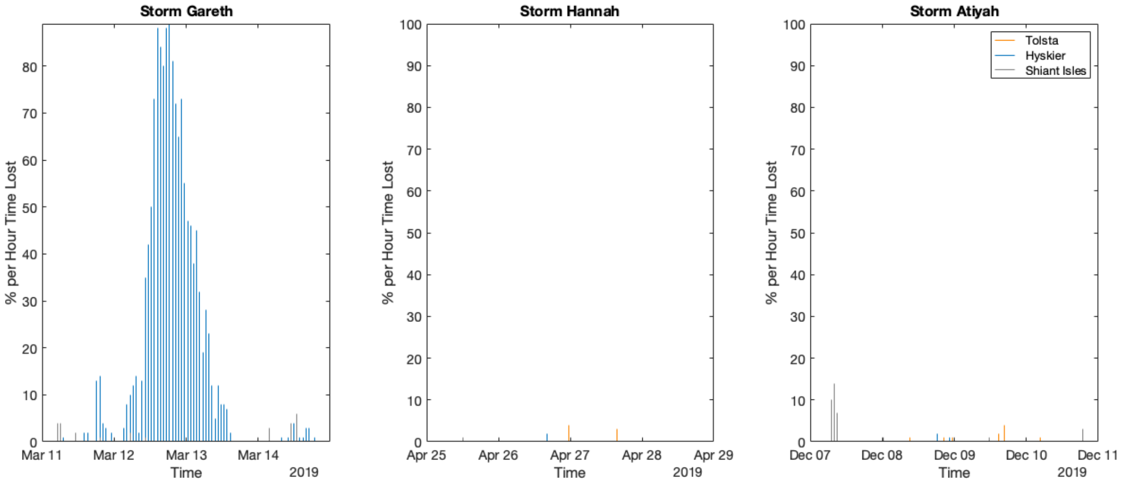

Supplementary Figure 2. The percentage of time lost on the C-POD recording platform during storm conditions, at Tolsta, Hyskier and Shiant Isles. The C-POD time lost is a result of ambient noise, sediment transport and data logger movement in the water column. During Storm Gareth, at Hyskier, the C-POD lost > 20% of recorded hourly data during peak storm conditions.

To quantify the temporal agreement between the manual labels and the two detection algorithms the Pearson corelation coefficient was computed (Supplementary Table 1). A correlation value closer to 1 indicates a strong positive linear relationship, meaning the algorithm closely tracks the manual labels. A value close to 0 indicates no linear relationship present between the two datasets. Where a negative coefficient is report, the detector outputs describe an inverse linear relationship to the manual labels. The approach allows us to objectively compare algorithm success at describing the temporal and diurnal patterns in delphinid presence. The Pearson coefficient was computed in Matlab using the hourly counts, which were summed for each hour of the day for the duration of each analysis period, per site.

Supplementary Table 1. Computed Pearson Correlation Coefficient metrics per analysis period for a) Tolsta, b) Hyskier, and C) Shiant Isles.

		**Jan**	**Apr**	**Jul**	**Sep**	**Nov**
**a) Tolsta**	CNN – Clicks Only	0.83	0.75	0.81		0.72
	CNN – Whistles & Clicks	0.83	0.68	0.75		0.85
	C-POD	0.77	0.63	0.66		0.65
**b) Hyskier**	CNN – Clicks Only	0.75	0.23	0.76	0.57	0.83
	CNN – Whistles & Clicks	0.74	0.15	0.76	0.82	0.93
	C-POD	0.66	-0.14	0.68	0.15	0.03
**c) Shiant Isles**	CNN – Clicks Only	0.21	-0.18	0.72	0.73	0.78
	CNN – Whistles & Clicks	0.33	-0.60	0.87	0.77	0.88
	C-POD	-0.30	0.06	0.55	0.50	0.41

Supplementary Figure 3 is to provide context regarding the anthropogenic chirp signal present in April at each of the three sites. The figure has been anonymised by removing the time and frequency axis of the spectrogram due to the sensitivity of the data. This figure provides insight into the similarity of these signatures to the whistle class, given their dominant energy occurs in an overlapping frequency range to delphinid tonal calls.

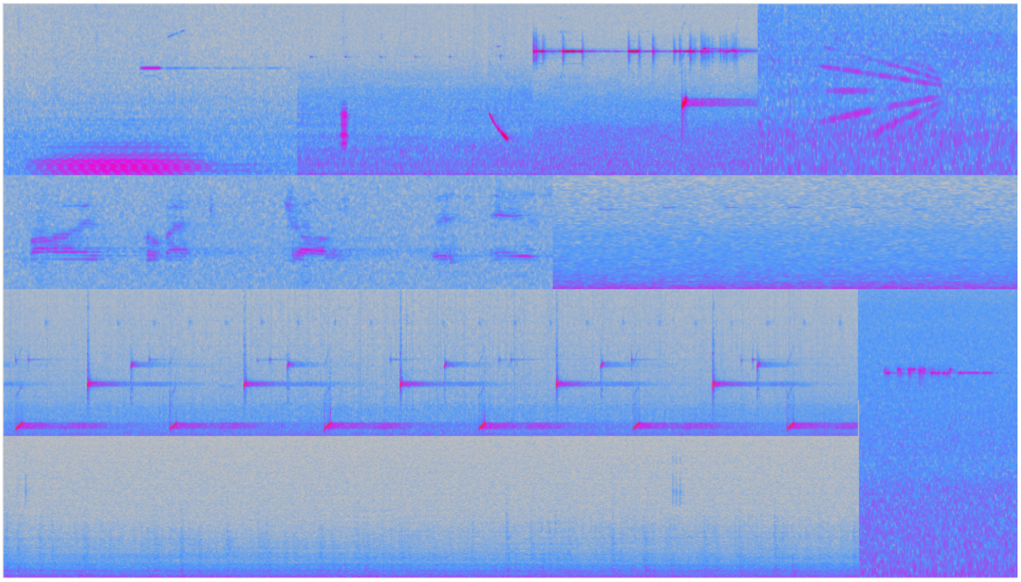

Supplementary Figure 3. Exemplar anthropogenic chirp signatures extracted from across the Scottish dataset. Spectrogram axis scales have been removed to protect the sensitivity of this data. Spectrograms are computed with a Hanning window, using a window size of 2048 samples and a 50% overlap.

Model Architecture.

The CNN used within this manuscript was developed previously, by the lead author and is published with Frontiers of Marine Science (White *et* al., 2022). A full explanation of the training data, network structure, and training regime used to develop the model is present within White *et al.,* 2022, but to provide context as to model relevant to this work a brief description is found below:

The network used in this work was built upon the EfficientNet model (Tan and Le, 2019), making use of transfer learning as a technique to harness the power of larger models. EfficientNet is a family of networks which use model scaling, balancing network depth, width and resolution to output state-of-the-art accuracies with relatively few parameters (Tan and Le, 2019). Our work utilized EfficientNet B0 the smallest of these networks. Using 5.3 million parameters it is 8.4× smaller and 6.1× faster than other commonly adopted architectures (Tan and Le, 2019). EfficientNet B0 is pretrained on the ImageNet database (Deng et al., 2009) with 1000 classes. The original EfficientNet B0 feature extractor layer is left frozen, the weights and biases determined through training on the ImageNet database were not updated during training. The final layers of the architecture, the classifier, have been replaced through fine-tuning and trained on the custom datset described within White *et al*., 2022.

The final classification layers are a set of fully connected layers, attached *via* a Global Average Pooling layer (GAP) which reduces the number of features to 1280. The GAP layer takes the

average of each feature map in the last convolutional layer of the EfficientNet B0 architecture and flattens the output of the feature extractor into a vector, which can be used as a feature descriptor and fed into the fully connected layers of the classifier. The data is passed through three fully connected layers with 512, 256 and 4 neurons in sequence. Between each layer are dropout layers of 50% and 20% respectively. The 512 and 256 dense layers use a ReLU activation function (Krizhevsky et al., 2012) The final four neuron fully connected layer classifies the input spectrogram into one of the four classes, using a softmax activation function. The softmax function outputs the pseudo-probability of an image belonging to each of the four classes and the network assigns a label based on the highest pseudo-probability value. The final model architecture had a total of 4.8 million parameters, of which 788,228 are trainable and 4,049,564 remain frozen during training updates.
