# Supplementary material for: Evaluating the performance of automated detection systems for long-term monitoring of delphinids in diverse marine soundscapes
Source: PLoS One. 2025 Jun 11;20(6):e0323768. doi: 10.1371/journal.pone.0323768 (PMC12157003; doi:10.1371/journal.pone.0323768)
Supplement: Attachment — Submitted filename: Response To Reviewers.docx [file pone.0323768.s003.docx]

Dear Editor,

We are grateful for the detailed and thoughtful reviews provided on our manuscript. The reviewers’ comments were comprehensive, and we respond to each comment below. We wish to stress that the changes to the manuscript are minor, and our major findings are unchanged. We believe that the manuscript has been improved by the review process and appreciate the thought-provoking comments provided by each reviewer.

Yours sincerely,

Ellen White

**Reviewer #1:**

***1.	Could the authors clarify the novelty of this study to highlight its unique contributions? Since the CNN model used here was originally designed in another work (citation [44]), is the novelty primarily in its application to a new dataset?***

The novelty of this work can be attributed to two points:

a) There has a been a significant focus on deep learning pipelines within underwater bioacoustics in recent years, but to date there has not been a study which evaluates their performance compared to a computationally cheap option of species monitoring. In this work we attempt to consider both approaches strengths and weaknesses to provide a commentary to the community on which method to choose depending on the research question at hand. Although the CNN chosen does not reflect the performance of a CNN trained under different conditions, in general our work presents the capabilities of a deep learning network, underpinned by the high up front cost with respect to manual labour and computing power, in contrast to the ‘plug and play’ approach to the C-POD. In addition, we draw attention to the fact that the CNN used in this work has been trained to identify a range of sound sources and is not specifically tailored to delphinid clicks (as a binary detector would be). We expect a delphinid click specific network would output even higher performance metrics, but this work sits as more of a baseline commentary on what a deep learning approach can offer the community, in contrast to what the C-POD offers.

b) Secondly, as you point out, the CNN within this work is being deployed on unseen data – data it has not seen during training, nor was the network fine-tuned prior to this work. We present performance metrics for the CNN within new acoustic environments as an ‘off-the-shelf’ approach to delphinid detection. We make it clear in the discussion that performance would improve if the user was to take this pre-trained model and adapt it to their own soundscape, but this requires some computational expertise. Therefore, the novelty here is the capability of an ‘un-adapted’ network to perform in a new acoustic environment, validated by the manual analysis, and outperforms an existing standard approach of species monitoring. The application of a network in an ‘off-the-shelf’ manner is not often displayed in the literature.

Overall, the unique contribution of this manuscript is to add to the wealth of knowledge available to researchers when planning the methodology of their study. To deploy a C-POD, or other automated detection instrument, or collect raw acoustic data and use an off-the-shelf algorithm for detecting signals within their data. The cost and storage needs for the latter far outweigh the former, but the data insights with the former are limited.

***2.	In section 3.1-3.4, the authors compare the CNN with Manual analysis and the C-POD, but several issues need attention:***

***In C-POD, the KERNO classifier detects click train based on parameters such as intensity, duration, frequency content and inter-click intervals (ICI). To ensure fair comparisons, the Manual Analysis and CNN should also output click train detections specifically.***

This raises an interesting point as to what constitutes a fair comparison. We seek to compare two approaches to delphinid detection, not to click train detection, since this is the more ecological relevant question. The fact that C-PODs make delphinid detections based on detecting click trains is inherent in their design, it should not constrain other systems or a manual analysis. We don’t believe that a fair comparison requires that the two approaches use the same reasoning to reach the conclusion of delphinid presence/absence.

The CNN used in this manuscript has been trained to detect delphinid clicks as a frame with three or more clicks present. To reduce false positives, during training the CNN is shown many acoustic frames which contain signals similar to delphinid clicks but originate from other sources, as well as single isolated click events, to learn that these are not part of the ‘click’ class. In this sense the CNN is in fact detecting click trains.

With respect to manual labelling of click events, the identification of clicks to be of delphinid origin as opposed to another source are based on the temporal and frequency characteristics of species present in Scottish Waters. The sampling rate of the SoundTrap is 96 kHz, meaning we cannot use the whole profile of the click to determine detections, but we can infer delphinid origin based on the ICI, frequency range and temporal nature of the signal. This was accounted for in the manual labelling process and a sentence has been added to the Manual detection methods section to make this clear to the reader:

Line 184 – 185: ‘Delphinid clicks were identified based on the temporal and frequency characteristics of broadband signals within the data, including the frequency range and inter-click-interval (ICI).’

***2.1 CNN: The authors state (Lines 168-169) that the CNN classifies chunks as containing delphinid clicks if the chunks include both click trains and burst pulses, but burst pulses are distinct from click trains. Additionally, the authors state on Line 175 that the CNN model prioritizes whistles over clicks. To align with the aim of detecting click trains, either the CNN model from [44] should be fine-tuned, or a new model should be trained specifically for the exclusive detection of click trains. Without this adjustment, the comparisons presented in Sections 3.1–3.4 lack validity. Furthermore, this adjustment could serve as a significant contribution or highlight the novelty of this study.***

Building on our response to the last question. The basis of the comparison is not as a click train detector, rather as a detector of delphinid presence/absence. The CNN classifies frames into one of four categories (whistle, delphinid click, vessel noise or ambient) and the L204 clarifies that burst pulses are grouped together with clicks.

The CNN approach is designed to output ecologically relevant information on delphinid presence, not to mimic the C-POD processing pipeline. The hierarchical training approach ensures that when multiple signals are present within a frame, the model prioritises detection based on temporal characteristics—first identifying whistles (as they are the shortest in duration), followed by clicks, and then vessel noise. This structure is necessary because the network is designed for single-label classification, meaning it assigns only one class per frame. While a CNN trained exclusively for click detection might yield higher performance metrics, the objective of this study is to demonstrate the broader capabilities of deep learning and highlight how an off-the-shelf network—without extensive fine-tuning—can still produce meaningful insights into species presence. Developing a separate binary click detector for comparison would not only be computationally demanding but would also detract from the novelty of this work.

***2.2 Manual analysis: (Page 8, line 151) The method for identifying click trains in the Manual Analysis needs further clarification. It would be helpful to align the manual analysis with the approach used by the C-POD -considering parameters such as intensity, duration, frequency content, and inter-click intervals (ICI), rather than relying solely on the number of clicks in a click-series.
Since the aim of this work is to develop an automated tool for extracting species presence, all types of clicks (e.g. click trains, burst pulses, buzzes, and regular echolocation clicks) can be used. Therefore, the C-POD should be tuned to detect all these signals, not just click trains, and the CNN model from [44] should be adjusted to prioritize click detection over whistles.
In summary, any comparisons should be made based on consistent outputs across the different methods to ensure validity and reliability.***

Regarding your first point on manual analysis for the identification of delphinid clicks using an approach similar to the C-POD, please see my detailed response to statement 2. A sentence has been added to line 151 to improve the clarification of the manual analysis process:

‘Delphinid clicks were identified based on the temporal and frequency characteristics of broadband signals within the data, including the frequency range and inter-click-interval (ICI).’

We have addressed in above your concern on the validity and reliability of the outputs across the different methods used in this work.

***3. The identification of vessels (both manual and CNN) is not utilized in this study. To avoid confusion for readers, please consider removing related descriptions.***

The reference to vessel detection has been removed from the manuscript, as you point out it is not utilised and will result in reader confusion. The following text has been removed from line 187: ‘and labellers also identified periods where vessels were present in the raw acoustic data’.

***4. This work lacks a background survey on the application of Deep Learning to marine mammal sound detection or classification. While the authors provide reasons for using CNN on Page 5, Line 83, could they also include examples of other state-of-the-art Deep Learning applications to further support their choice of CNN.***

To further support the use of a CNN for a marine mammal detection and classification problem I have included examples of Convolutional neural networks to other marine mammal species beyond delphinids to emphasise the success of these architectures within this domain. I have also added in a reference to Dan Stowell’s 2022 roadmap for deep learning in the bioacoustics community which eloquently lays out the performance benefits of a CNN compared to other machine learning techniques.

Line 103, on page 5 now reads: One widely adopted ML approach makes use of Convolutional Neural Networks (CNNs) which were originally developed for image processing, but have been used to classify acoustic data and have achieved high accuracies when applied to the task of detecting marine mammal vocalisations[42 - 46], including dolphin vocalisations within wideband PAM data recorded in variable marine environments [47 – 51], compared to other state of the art machine learning techniques [52].

The papers now referenced from [42-46] are:

Bermant, P.C., Bronstein, M.M., Wood, R.J., Gero, S. and Gruber, D.F., 2019. Deep machine learning techniques for the detection and classification of sperm whale bioacoustics. Scientific reports, 9(1), p.12588.

Shiu, Y., Palmer, K.J., Roch, M.A., Fleishman, E., Liu, X., Nosal, E.M., Helble, T., Cholewiak, D., Gillespie, D. and Klinck, H., 2020. Deep neural networks for automated detection of marine mammal species. Scientific reports, 10(1), p.607.

Allen, A.N., Harvey, M., Harrell, L., Jansen, A., Merkens, K.P., Wall, C.C., Cattiau, J. and Oleson, E.M., 2021. A convolutional neural network for automated detection of humpback whale song in a diverse, long-term passive acoustic dataset. Frontiers in Marine Science, 8, p.607321.

Rasmussen, J.H. and Širović, A., 2021. Automatic detection and classification of baleen whale social calls using convolutional neural networks. The Journal of the Acoustical Society of America, 149(5), pp.3635-3644.

Ruiz, M.R., Rossi, C. and Esteban, J.A., 2023. Fin whale pulse detection with deep neural networks. Ecological Informatics, 77, p.102243.

***5. Abstract: please consider merging the two paragraphs into a single cohesive paragraph to provide a concise summary of the work.***

I have removed the paragraph spacing in the abstract as requested.

***6.	page 3, line 44: missed a period after ` offline systems’.***

Corrected.

***7. Page 4 lines 56-57: Does this sentence means: the C-POD detects individual sound clicks while operating (on-line), and later, during offline processing, those clicks are grouped together into click trains based on their characteristics.
The authors mention that the C-POD performs online detection; however, the sudden introduction of offline processing could confuse readers. It would be helpful to provide a clearer explanation of the processing steps involved in the C-POD detection workflow, including how and when the transition from online detection to offline processing occurs.***

For clarity I have edited lines 77 -78 to read ‘Onboard the C-POD autonomously detects the presence of clicks between 20 – 160 kHz in real-time, storing summary data which is used to link detected events into species specific trains during post processing.’

***8. Page 4, line 60: There are two ‘for which’ at the beginning of this line.***

Corrected: I have now removed the second ‘for which’ from line 80.

‘Proprietary software containing a custom classifier (KERNO) classifies clicks into trains (regularly spaced series of similar clicks) based on their intensity, duration, frequency content and inter-click intervals (ICI), **for which** three classification filters are available: high-, medium-, and low-quality detections [31, 32].’

***9. Considering adding a sentence at the end of the Introduction to outline the structure of the manuscript. This will help guide readers through the subsequent sections.***

After careful consideration I feel the introduction should remain as is, with the final paragraph alluding to the research questions we will answer without giving the reader too much information that may yet be relevant to their understanding of this work, see lines 118 – 123:

‘In this work we conduct the first empirical evaluation of the performances of a CNN and the C-POD on the task of delphinid detection within PAM data recorded off the west coast of Scotland (Figure 1). An open-source multi-sound source CNN [44] is used without any network adaptation to the test data. We compare the output of both algorithms, across diverse acoustic conditions, against manually labelled recordings to evaluate their suitability in the standard processing toolkit for long-term delphinid monitoring.’

However, I have added the following sentences to the methods section and the results section:

Methods (125:128):

‘This section outlines the methodology use, beginning with a detailed summary of the data acquisition process (Section 2.1). Section 2.2 describes the analysis of acoustic data through each approach: a) manual, b) the CNN, and c) the C-POD. Finally, Section 2.3 introduces the evaluation metrics used in subsequent sections to assess the performance of each approach.’

Results – Line 261:

This section evaluates the performance of the C-POD and the CNN, applied to broadband acoustic data, in detecting delphinid presence across diverse underwater soundscapes using the manual labels as groundtruth. This analysis is based on 3,000 hours of acoustic data collected throughout 2019 in western Scottish waters. First, we conduct an overall comparison of click detections (Section 3.1). The output of each detector is then examined to identify temporal and seasonal patterns (Section 3.2). Sections 3.3 and 3.4 compare detector performance across varying ambient noise conditions using ROC curves and precision-recall metrics. Finally, Section 3.5 incorporates whistle detections output by the CNN to evaluate the benefits of monitoring delphinids using multiple signal types.

***10. In Figure 2 or in the text, please provide an example of the labels used for CNN, similar to the numeric labels presented for Manual Validation and C-POD. This would ensure consistency and help readers better understand the comparison.***

I have amended the figure legend of Figure 2 to read: ‘Schematic depiction of the data collection and subsequent analysis workflow used for the determination of delphinid detection positive hours (dph) by a) Manual, b) CNN, and c) CPOD detection. Manual analysis applies a positive or negative label to the presence of delphinid clicks and delphinid whistles. The CNN outputs one label per input frame; Ambient noise, delphinid whistle, delphinid click or vessel noise, and the total frames per class are summed per file to provide a binary positive or negative presence of clicks per hour. The C-POD outputs detection positive minutes for delphinid clicks, for each minute of the recording hour, with the total sum of positive minutes converted to a positive or negative detection positive hour.’

***11. Page 8 line 157: Please provide a detailed description of the CNN architecture used in this work. Relying on references to another paper for such essential information could hinder readers' understanding of your methodology.***

After careful consideration of this point, I do not feel it is appropriate to provide a detailed description of the CNN architecture used within this work, particularly as it is published within other manuscripts (White *et al*., 2022 & White *et al*., 2023), and to reproduce it in detail would add substantial information to this manuscript which is unnecessary for non-expert readers.

The pre-processing of the acoustic data required for input to the CNN is described in detail in this manuscript, and we feel this is satisfactory content for a reader to comprehend our work and for it to be reproducible.

I have edited the main text (Line 165: 169) to read: ‘The details of the network architecture for the CNN used in this task can be found in White et al [44], with additional information available in the Supplementary Material. The original network, built upon the EfficientNet B0 architecture, was trained on acoustic data recorded in the COMPASS array during 2017 and 2018.’

***12. The authors have provided the threshold for both whistle and click detections, but have not specified the threshold used to determine the presence of clicks. Please include this information for clarity.***

I apologise for the confusion around thresholding the click and whistle detections. The threshold used for whistles and clicks in section 3.5 is 0.9 and the manuscript has been edited to clarifying this (Line 231: During post-processing a threshold can be applied, so that only labels with a detection score above the threshold are valid when computing dph. A threshold of 0.9 is applied in Section 3.5 of this manuscript). However, in the raw comparison of click detections from the CNN vs the C-POD (section 3.1 – 3.4) no threshold is applied. This is to provide continuity with the C-POD for which we allowed all detections to be counted (high, med, and low – Line 185), and to explore performance of the CNN at specific thresholds with respect to evaluation metrics (sections 3.3 & 3.4).

***13. Page 8 line 174: Please clarify the meaning of ‘frame’ in the context of this work. Is it synonymous with ‘chunk,’ or does it refer to a different concept?***

To provide consistency I have removed all references to ‘chunk’ in the manuscript, using the term ‘frame’ to refer to a segment of audio data spanning 3-seconds in duration, each frame is computed as a three-channel spectrogram and fed into the CNN for processing.

***14. Page 9, lines 182-186: Are there existing statistics in the literature regarding the performance of the C-POD in detecting click trains? If so, please include this information to strengthen the rationale for selecting this method for comparison.***

The methodology described in lines 236 - 240 presents the standard analysis pipeline for post-processing C-POD data as provided by the manufacturer. The paragraph cites Nuuttila et al., 2023, Acoustic detection probability of bottlenose dolphins, with static acoustic data loggers in Cardigan Bay. This paper is co-authored by the manufacturer of the C-POD and outlines the standard approach to classifying delphinid click trains from the onboard detection data. Dolphins cannot be rigorously monitored by isolated click events; due to the possibility they occur from a multitude of underwater sound sources. Grouping them into click trains is an essential part of the analysis pipeline and is central to the C-PODs operation.

To strengthen the literary support for this statement, and highlight existing work on the performance of the C-POD at detecting click trains I have also included a reference to [30] & [31] – Todd et al., 2023, What the F-POD? Comparing the F-POD and C-POD for monitoring of harbor porpoise & Sarnocinska et al., 2016, Comparing the performance eof C-PODS and SoundTrap/PAMGuard in detecting the acoustic activity of harbor porpoise. These papers outline a similar methodology for identifying click trains with the C-POD, supporting our approach, and were referenced elsewhere in the manuscript.

***15. For Table 2, please consider adding an additional row to show the overall number of hours for each period. This would provide a clearer summary of the data.***

Thank you for your recommendation. To provide a clearer summary of the data to the reader I have included the total number of analysis hours per site as the first data column of Table 2. To enhance readability, I have also shortened the column titles to condense the scale of the table. Column headers are now explained in the table caption. Table 2 is now formatted as:

**Table 2**. The total true positive (*N_TP_*) and true negative (*N_TN_*) dph for delphinid clicks during the manual analysis of each temporal period and site. Temporal periods are defined as: Jan – January (24^th^ – 31^st^), Apr – April (1^st^ – 7^th^), Jul – July (1^st^ – 7^th^), Sep – September (1^st^ – 7^th^), Nov - November (17^th^ – 23^rd^) , St. G – Storm Gareth, St. H – Storm Hannah, and St. A – Storm Atiyah.

| **Site** |  | **Total**  **Hours** | **Overall** | **Jan** | **Apr** | **Jul** | **Sept** | **Nov** | **St. G** | **St. H** | **St. A** |
| --- | --- | --- | --- | --- | --- | --- | --- | --- | --- | --- | --- |
| Tolsta | $N_{TP}$ | 984 | 452 | 122 | 33 | 56 |  | 116 | 33 | 22 | 70 |
|  | $N_{TN}$ |  | 532 | 70 | 135 | 112 |  | 52 | 63 | 74 | 26 |
| Hyskier | $N_{TP}$ | 1152 | 313 | 33 | 10 | 52 | 57 | 115 | 7 | 5 | 34 |
|  | $N_{TN}$ |  | 839 | 159 | 158 | 116 | 111 | 53 | 89 | 91 | 62 |
| Shiant Isles | $N_{TP}$ | 864 | 298 | 39 | 43 | 46 | 93 | 77 |  |  |  |
|  | $N_{TN}$ |  | 566 | 153 | 125 | 122 | 75 | 91 |  |  |  |

***16. Page 10 line 215: The sum of 532, 839 and 566 equals 1937, not 1936. Please double-check this calculation and verify all numerical values in tables for accuracy.***

My sincere apologies for this oversight. I have now corrected this line to read: ‘The overall total dph is 1063, with 1937 hours without any delphinid detections made, for an imbalanced dataset of roughly 2:1 in favour of no detections.’

I have also checked the remaining calculations and am confident there are no other computational errors within the manuscript.

***17. Page 10 line 203: The description of FPR in this line is incorrect. FPR refers to the proportion of actual negatives that are incorrectly classified as positives. Please revise this definition for accuracy.***

I apologise for the inaccuracy in this statement, of course the false-positive rate is infact the proportion of positive detections which are incorrect, output by each methodology. This line has been revised to correctly read: The false-positive rate (FPR) is the proportion of the positive detections that are **incorrect**, and the false-negative rate (FNR) is defined equivalently for the negative detections:

***18. Page 11 lines 218-219: Could the authors clarify how the proportions of dph were calculated? Please also double-check that all proportions provided are accurate.***

The following method was used to calculate the proportion of dph at each site: For each period in Table 2 e.g. Overall at Tolsta, the proportion was calculated by dividing the number of true positive hours (N_TP_) by the total number of hours (the sum of N_TP_ + N_TN_). This proportion was then converted to a percentage by multiplying by 100, representing the percentage of true positive hours, relative to the total observation period, identified by the manual labelling.

E.g. Overall proportion at Tolsta:

Total_hours = (N_TP_ + N_TN_)

Total_hours = (452 + 532)

Total_hours = (984)

Proportion = (N_TP_ / total_hours) * 100

Proportion = (452 / 984) * 100

Proportion = 45.930 🡪 rounded to 46%

For specific periods, where we describe the proportion of dph during storm periods the same method was followed, however the total observation hours is the total hours at Hyskier for Storm Gareth + Storm Hannah + Storm Atiyah, with the total N_TP_ summed for the three sites. To improve clarity on this I have included the breakdown of storm specific dph proportions within the main text:

‘With respect to site, Tolsta experiences the greatest proportion of dph (46%) and Hyskier the lowest (27%), with the effect of storms most notable at Hyskier with only 19% of hours positive detections for delphinid presence across the three periods (Gareth = 7%, Hannah = 5%, and Atiyah = 35%, Table 2).’

The percentage proportions here are to present an easy overview of the data to the reader, with all values a simple calculation from the information present in Table 2. I manually checked all of the proportions in this paragraph and am confident in their accuracy. To confirm this, I have provided the raw percentage values computed for each seasonal period, and overall for all three locations in a table below.

|  | **Tolsta** | **Hyskier** | **Shiant Isles** |
| --- | --- | --- | --- |
| **Overall** | 45.93 | 27.17 | 34.49 |
| **January** | 63.54 | 17.19 | 20.31 |
| **April** | 19.64 | 5.95 | 25.56 |
| **July** | 33.33 | 30.95 | 27.38 |
| **September** | NaN | 33.93 | 55.36 |
| **November** | 69.05 | 68.45 | 45.83 |
| **St. Gareth** | 34.38 | 7.29 | NaN |
| **St. Hannah** | 22.92 | 5.21 | NaN |
| **St. Atiyah** | 72.92 | 35.42 | NaN |

***19. Page 15 line 327, page 16 line 333: In the parenthesis on both lines (Table, Figure 5), please specify which sub-figure in Figure 5 is being referred to for the discussion to ensure clarity.***

References to figure 5 within the main text have been corrected, to point the reader specifically to the sub-plot described in the text.

In line 417, the text has been amended to read: ‘At Tolsta the CNN produces a higher rate of true positive detections, with a comparative false-positive rate to the C-POD in both weather conditions (Table 4, Figure 5a,b & c).’, as we are describing the overall results of TP detections compared to FP detections for all locations.

In line 426 the reference has been edited to be specific to sub-plot b, describing the performance at Hyskier: ‘At Hyskier the C-POD fails to detect any of the dph during storm conditions, and only correctly identifies 7% of the dph in non-storm conditions, which results in a low FPR impacting the precision and TPR scores (Table 4, Figure 5b).’

***20. Page 16, line 345: the authors mention that the CNN generated a single label for each audio data chunk. However, on page 8 Line 168, it is stated that the network classifies each chunk into one of four labels: ambient noise, delphinid whistles, delphinid clicks (click trains and burst pulses), or vessel noise. This inconsistency is confusing and needs clarification.***

To clarify we have changed L345-348 to read: “The CNN used in this work generates a single label for each frame, labelling it as either, delphinid whistle, delphinid click, vessel noise or ambient noise. Of these, both of the first two classes indicate delphinid presence, in this section we consider the impact of including data from the whistle class as well as the click class (Figure 6).”

***Reviewer #2: The paper "Evaluation the performances of automated detection systems for long-term monitoring of delphinids in diverse marine soundscapes" is well written, interesting and with a solid conclusion. It is a useful paper that will help scientists and conservationists in their choice of a monitoring device.***

***However, I have one serious objection (why say you are only comparing detection software and not the recording device?), and think the presentation of the manuscript could be improved to better the general clarity of the work, and help further the future users of these instruments. I will first mention some general suggestions and then go into detailed comments.

***** General comments :
* On the general purpose (and title) : to my judgement, the comparison not only involves two detection techniques, but also two recording devices - it is difficult to separate them, given the integrated nature of the C-POD . CNN analyse on data gathered with a poor recording device would probably have a different efficiency. This 'objection' does not lessen the utility of the work, it's rather a suggestion that the study should clearly mention hardware as well as software in the comparison of the performance.***

Thank you for raising your concern about framing this work as a comparison between detection techniques. Please note the title is “Evaluating the performance of automated detection **systems**….”, this was carefully chosen as “system” encompasses hardware and software. We do clearly mention the hardware, as described in lines 146 – 149 indicating to the reader that the raw audio data is collected onboard a SoundTrap 300HF.

We believe the confusion arises because of the terminology we use, one system we call “CPOD” and the other system we refer to as the “CNN” whereas what we mean is a CNN applied to raw acoustic data. We have tried throughout the manuscript to reframe the use of ‘CNN’ as a term to be a specific reference to the software/detection algorithm rather than a hardware+software unit which describes the C-POD. I do think that the detail in lines 216 – 224 lay out the methodology regarding feeding raw acoustic data (with pre-processing applied) to the CNN to get a detection file output with labels and detection scores. For that reason I think it is clear that CNN is used in respect to the processing algorithm, and will be effected by the quality of input acoustic data.

**** Use of metrics : the metrics used are generally well defined (with some exception, see detailed comments), but may be to numerous, at the risk of losing the reader in a forest of parameters without always explaining the interest of each. I would globally advice to use fewer metrics and stick to them in all comparisons, justifying which is interesting in each case. This is already partly done in the paper but could maybe be more systematic.***

I appreciate your feedback on the numerous metrics we report in this manuscript, also highlighted by reviewer 1, I have reduced the number of the metrics used throughout. I have expanded the evaluation metrics section to make it clear which metrics report the same thing (or very similar). Where appropriate I do use a variety of terminology, in order to interpret the results for each specific sections. For example, The F1-score is not relevant to the interpretation of overall performance where I discuss ROC curves and Precision and Recall curves, and it is often clearer to state ‘missed detections’ instead of 1-Recall. I think the overall clarity of the results section has greatly improved by scaling down the reported metrics, while maintaining a critical quantitative analysis of both systems performances.

**** The comparison is well detailed in the 'results' part (maybe with too much details?) but very few explanations are given to explain the trends. The high level of false positives in the whistle detection is explained by a deeper insight into the manually revised data. The same should be done for the other main results: why is the C-POD so little sensible ? Can it simply be because of the recording device sensibility (and not the detection software) ? From my experience, individual C-POD can have different sensibilities, especially if 'old'. You could perhaps check the signal to noise ratio of the manual detections in some cases of lost information, for instance. At high frequency, the level of noise is sometimes low and sensibility of the recording device can be quite important. I would maybe suggest that 100 m is rather deep for click detection, given the directionality of the clicks and of the sensors at high frequency ?***

Thank you for commending our detailed analysis on the whistle detection, and I have made efforts to further explain the results as you will see addressed throughout your specific comments. The internal working of the C-POD is opaque, and one can only speculate as to why detections are missed, which we have done. We hope our paper highlights that where a false positive detection is harmful the C-POD is a very useful tool, due to its conservative nature, however it does miss many detections. For specific monitoring cases this may not be relevant, for other general monitoring purposes it may be more important to know the presence of animals with limited focus on false positives, in which the CNN excels. The interpretation of the two systems weaknesses is slightly beyond the scope for this paper as often it will be environmental and contextual information that effect their performance, rather we are showing when all of these are controlled for how they compare to a manual approach to species identification.

Please note three different C-PODs and SoundTraps are used in the data collection, and this is now made clear in Section 2.1, lines 146 – 149.

I appreciate your comment regarding the depth of the sensors at 100m, however this paper considers the relative performance of the two systems. They were both deployed at 100 m, so would have been exposed to the same number of clicks, allowing us to make an appropriate comparison.

***The figures quality should be improved (maybe some of them could be skipped without changing the quality of the paper).***

The submitted figures are of a higher quality.

**** Abstract :
l.4 "the work compares two methods" : as already mentioned in the general comment, I think what is compared is "recording device + method", the two cannot be separated. Thus I would mention the Soundtrap device as part of the general result.***

The abstract has been edited accordingly.

*** ***Introduction*** *:*
***l.25 ref (1) does not seem to be exactly to the point ?***

Thank you for pointing this out, this was an incorrect reference and has been corrected.

***l.26 "to effectively protect marine these species": not very clear***

The sentence has been corrected to read ‘In order to make informed decisions about existing practices and develop new mitigations to effectively protect marine species, policymakers require reliable sound scientific information regarding species habitat use and distribution’

***l.27 ref(3) is about invertebrate and noise , not really habitat use and distribution ?***

This reference is for the broader use of sound for monitoring species habitat use and distribution, as well as the impact of underwater noise on species, so has been retained.

***l.34 ref (15) and (16) are only about signature whistles, it could be more general to illustrate your point ?***

Thank you for highlighting the specificity of these two papers to the whistle repertoire of delphinid species.

In line 49 [15] is now a reference to Janik, V.M., 2009. Acoustic communication in delphinids. Advances in the Study of Behaviour, 40, pp.123-157. In lines 50 – 53 [16] has been changed to Au, W.W.L., 2004. Echolocation signals of wild dolphins. Acoustical Physics, 50(4), pp.454-462

The original references remain in the list at the end of the paragraph to support the broad statement made there.

***l.35 "signals between 20-100 kHz" : what about narrow band high frequency (NBHF) delphinids ? Their clicks go up to 135 kHz or more ... Even if you are not concerned in Scotland, the definition should not exclude them.***

We have amended the frequency range quoted here to be ‘ highly directional transient signals between 20 – 140 kHz known as echolocation clicks’.

***l.44 "systems Off-line" -> "systems. Off-line "***

Line 44 has been edited to include the necessary punctuation.

***l.52 "C-POD" presentation : you should definitely explain here that C-POD are outdated, and mention F-POD. Since a lot of scientists or ONG still use C-POD, it takes nothing away from the interest of your study.***

Line 54 has been changed to read:

‘Although now superseded by a new model, the F-POD (full waveform capture pod) [26], the C-POD continues to be used as an integral tool within global monitoring programs for assessing spatio-temporal patterns of species occurrence [26 – 30] due to its capability for low-cost long-term continuous monitoring.

***l.94 the CNN has been trained (partly) on data from the same project in one of the places (Tolsta), hasn't it ? You should maybe mention this here.***

The CNN was originally trained with acoustic data recorded at Tolsta during November of 2017 (White et al., 2022). The data used within the manuscript was recorded in 2019 and makes use of three locations. I think to add a discussion of this at line 94 would be confusing to the reader as we have yet to lay out the methodology of this work including recording sites and analysis periods.

The following has been added at line 214 ‘The acoustic data used for training the system were collected in completely different years from one of the sites in this study, and there was only one site in common, specifically Tolsta, see White et al., (2022)’.

* **Methods :
*l.113 In my copy, fig 1 has a very low quality.***

The submitted figure quality is much higher.

***l.143 In my copy, fig 2 has a very low quality.***

The submitted figure quality is much higher.

***l.160 cf my comment on line 94***

See correction regarding L94

***l.170 : I couldn't see where you mentioned what probability threshold you use, and why (because it was the one you used in your previous work?). At the end you mentioned a 0.9 threshold, but I seem to understand it was not your first choice ?***

I apologise for the confusion around thresholding the click and whistle detections. In the raw comparison of click detections from the CNN vs the C-POD (section 3.1 – 3.4) no threshold is applied. This is to provide some consistency with the C-POD for which we allowed all detections to be counted (high, med, and low – Line 185), and to explore performance of the CNN at specific thresholds with respect to evaluation metrics (sections 3.3 & 3.4).

The threshold used for whistles and clicks in section 3.5 is 0.90, and the manuscript has been edited to clarifying this in line 244 of section 2.2: ‘In post-processing an additional threshold was introduced to the CNN, to allow the user to control the trade-off between false alarms and missed detections when computing the dph. This acted so that only detections above the threshold were considered when determining dphs . A threshold of 0.9 is applied in Section 3.5 of this manuscript’.

***l.175 "where whistles were prioritised over clicks due to a shorter temporal length" : I don't really understand this sentence.***

This is has been edited to read "where whistles were prioritised over clicks".

***l.177 "a dph determined for ..." -> "a dph is determined for ..."***

Line 177 has been corrected.

***l.179 which threshold ?***

The text in L178 & L179 has been changed to read: ‘In post-processing an additional threshold was introduced to the CNN, to allow the user to control the trade-off between false alarms and missed detections when computing the dph. This acted so that only detections above the threshold were considered when determining dphs. A threshold of 0.9 is applied in Section 3.5 of this manuscript’.

***l.193 and supplementary material : OK***

No correction requested

***l.198 : "These calculation use the manual labels as ground truth" I would mention here the remark of l. 384 and following, that the "ground truth" is counted only on 1/3rd of the total time ! It is rather counter-intuitive, though rather well justified in l.386.***

Thanks this is a helpful addition, and I have changed the text in line 215 to: ‘This is a practical advantage that the C-POD offers over recording and processing raw acoustic data, so it is reasonable to maintain this benefit of C-PODs, although the ground-truth labels account for only one third of the C-PODs recording period’.

***l. 199 : I would mention (since these are notions that you will use later) that Recall is also called Sensitivity and it is also the TPR (True Positive Rate)***

I have improved the Evaluation Metrics (section 2.3) in line with your feedback. I have edited Lines 223 – 225 to read:

‘To quantify the performance of each detection method Accuracy (*A*), Precision (*P*), Recall (*R*) and the F1-score were computed (equations 1 – 4). We note that Recall is also referred to as the True-Positive Rate (*TPR*) within this manuscript. These calculations use the manual labels as ground truth.’

***l.203 : "the FPR is the proportion of the positive detections that are correct" is uncorrect***

Thank you for pointing out the error this statement. This now reads ‘the FPR is the proportion of the positive detections made that are incorrect’.

***l.204 : FNR should be (better) explained. It is also 1-Recall, which means, I think, that it is not very useful to mention it along with Recall.***

The explanation of the false-negative rate has been improved within line 231, and I have made clear the relationship of the FNR to Recall.

The false-positive rate (FPR) is the proportion of the positive detections that are incorrect, and the false-negative rate (FNR) is defined equivalently for the negative detections. The false negative rate is the rate of missed detections and is equal to 1 - Recall:

$$FPR= \frac{N_{\mathrm{FP}}}{N_{\mathrm{FP}}+ N_{\mathrm{TN}}} ,$$

(5)

$$FNR= \frac{N_{\mathrm{FN}}}{N_{\mathrm{FN}}+ N_{\mathrm{TP}}} = 1-R.$$

(6)

***l.208 : "manually" -> "manual"***

Corrected.

**** Results
l.211 : Mention (in the 3.1 title?) that here you are comparing click detection only (and not delphinid detection). It is written in l.213, but not very obvious when you read it for the first time***

I have changed the section title of 3.1 to ‘Click Detection Evaluation’.

***l.221 : table 2 : you could maybe mention the % of TP, to make the reading easier (E.g. N_{TP} = 452 (46%) )***

In line with feedback from Reviewer 1, Table 2 has been amended to include the overall number of analysis hours per site. To improve this further I have added the percentage overall of true-positive hours per location to the table. I have avoided including the percentage true positive hours for each specific season as I feel this would introduce too much information to an already numerically dense table. The computation of percentages from the values in this table are straightforward and used only as a reference for understanding the distribution of ground-truthed labels.

***l.226 : it is interesting to mention the processing time of each method. CNN has been trained in another study but with the same kind of data and environment, maybe you should mention it? It would be interesting, also, to mention the volume of data for each case? Also, maybe give directly the total time, not "by week of data" so the reader doesn't have to look back how many weeks you had and do the multiplying for himself ;)***

We have added into Table 1 the total size of the raw acoustic data, 600 GB for all 3000 hours, and provided the total size of each data set per site (226 Gb at Tolsta, 199 Gb at Shiant Isles, and 265 Gb at Hyskier). The following text has been added into the Analysis section of the methods c) C-POD detection: The total size of extracted data from the C-POD was 2Mb, totalling 650 Kb per site for all analysis weeks. This is a significant reduction in data volume compared the raw acoustic data necessary for the CNN (Table 1).

With respect to processing times I have included the following information within the methods section for each respective system/algorithm:

C-POD: Total processing time to extract detection positive hours from the C-POD was one hour for all sites and seasons.

CNN: Overall, the CNN took 30 hours to process the broadband acoustic data for all sites and season, averaging 1.5 hours per week of data. Across the three sites this amounted to 10.5 hours for Tolsta, 7.5 hours for Shiant Isles, and 12 hours for Hyskier.

***l.231 : C-POD in this case has a high precision but low recall (more classically paired with precision than FNR)***

In line 272, the reference to FNR has been changed to include recall, as well as highlighting the missed detection. I have edited the text to read: ‘From Table 3 it is evident that the C-POD’s performance is characterised by a low level of false positives (overall 2% for the C-POD compared to 7% for the CNN), which results in a correspondingly high precision for the CPOD (0.96, Table 3). However, this is paired with a low recall score of 0.47 and a large number of missed detections, specifically a *FNR* of 53% compared to 30% for the CNN.’

***l.238 : FNR is 1-R so it is not really indispensable to put it in this table, which is already rather large.***

I have removed the FNR column from Table 3.

***l.247-250 : a relevant comment***

***l.263 : In my copy, fig 3 has a very low quality.***

The submitted figures are of a higher quality

***l.265 : why smooth the data ?***

The data has been smoothed in these plots to make the trends more visually evident.

***l.275 : "correlation was weakest at Shiant Isles" : could you explain why (or in the discussion part) ? It seems very low indeed.***

We hypothesize that the low correlation coefficient observed at Shiant Isles is a consequence of the weak seasonal trends observed in the dph data compared to the other sites. However, the specific environmental context behind the results of each specific site is slightly outside of the scope of this work, as the ambient environment anywhere will impact recorder / detection system performance. They key result at Shiant Isles is if both detectors performance declines, or how they compare to each other. We note that during the manual analysis SNR of echolocation clicks at Shiant Isles was relatively lower than at other locations, but the mooring location itself proves to possess a variable soundscape presenting a difficult acoustic environment for any detection pipeline.

***l.291 : TPR has not been defined (=R)***

The true-positive has now been defined in section 2.3

***l.293 : FNR is a bit redundant here, isn't it (=1-TPR = 1-R) ?***

Although I agree with your sentiment, I have decided not to remove the FNR column from Table 4. Where appropriate the false negative rate is used to describe important trends in the data. The table includes TPR, which is equal to Recall (and I have edited the table caption to highlight this), however this section heavily focuses on the rate of missed detections between the two algorithms. To remove this column would require the reader to manually do a subtraction for each FNR value using TPR from Table 4.

***l.296 : In my copy, fig 4 has a very low quality. You could use a more standardized scale (0-1, except maybe for FPR which is usually low in unbalanced populations).***

The submitted figures are of a higher quality

The axis scales have been purposefully selected to demonstrate the relationship between each detector’s performance. Neither preform perfectly, and so a 0-1 scale albeit standard, will make the specific trends more difficult to identify.

***l.311 : Could you maybe justify why you chose these metrics, not the same as before? Precision was not considered useful in this case ?***

The use of ROC and precision-recall curves were used to directly compare the performance of each method against the other. By using the ROC curve and the P/R curve we can isolate the point at which performance is comparable in terms of one metric and compare the performance in terms of the other, which is a common reason for constructing such curves.

***l.320 : In my copy, fig 5 has a very low quality. I would definitely use the same scales for the different sites.***

The submitted figures are of a higher quality

I have preserved the scaling’s as in the original submission. Because the purpose of this figure is to compare the performance of each methodology within a location and not to draw comparison between sites. Fixed scales between sites hinders the comparison between methods.

***l.329 : Storm may not be of great influence on the performance of the devices then ? Did you check in the data to see how 'storm' was translated into noise? White noise, or coloured, constant, or with interruptions? It's not obvious how a storm is recorded at more than 50 m deep.***

The fact that storms cause noise at the surface through wave agitation, and weather (wind, rain) is well established. Further, underwater ambient noise levels in this band are known to be highly correlated with surface wind speed (e.g. Wenz, Gordon M. "Acoustic ambient noise in the ocean: spectra and sources." *The Journal of the acoustical society of America* 34.12 (1962): 1936-1956). Noise is very different from the hydrodynamic effect of the storm, whereas the hydrodynamic effects may only influence the top few 10’s m of the water column, noise will propagate to great depths, certainly 100 m.

***l.331-338 : It seems to me the comparisons here are a bit confusing, you are using different metrics for the different methods.***

To improve the clarity in the comparison drawn here between the two methods I have rephrased this section to read:

‘The CNN outputs a notably high FPR of 0.12 compared the C-PODs 0.01, however the CNN outputs a true-positive rate of 0.50 which is significantly higher than the C-PODs true-positive rate of 0.12 (Table 4, Figure 5c), indicating a difficult soundscape for classification.’

***l.340 : this conclusion seems rather in contradiction with the sentence of l.328-329***

There is no contradiction. L328-329 says there is no change in the performance of the detectors relative to each other. Whereas L340 says their absolute performance is affected.

***l.349 : here, the threshold is mentioned.***

See correction for L170

***l.343 : General comment on part 3.5 : This part is rather short compared with the previous one and could maybe be a little more detailed (or removed altogether if you want to stick to clicks detections). The very high numbers of false positives, not only in April (table 6) are rather shocking (they tend to claim that dolphins were present 88 % of the time in overall, with 100 % in nov in 2 sites). During the storms, the number is especially high, triplicating the detections). This result should be addressed in the first place, I think, with the other comments coming on later. Why none of the metrics that were previously described are used in this part ?***

Thank you for your feedback on section 3.5. To improve the emphasis that this section is focused only on CNN performance I have edited the title of section 3.5 to read ‘Performance of the CNN based on both clicks & whistles’. I feel it is important to evaluate the CNN with respect to the influence of whistles, as the network is trained with a priority on these signals. To describe the metrics in detail as with the other sections is not necessary as we are not comparing these results to the C-PODS – rather we are demonstrating the influence of whistles on the CNNs ability to identify ecological trends in delphinid presence. To discuss in detail the metrics of whistle detection would require further analysis which we feel is out of the direct scope of this paper.

In the discussion, at line 475 I have added the following text: ‘We advise users to make use of the CNN output on a sub-sample of data to visualise and explore signals which may lead to missed detections, or false positives, ensuring that any efforts to fine-tune the network to the study site are grounded in robust data exploration. This is a notable strength of any CNN pipeline over a data logger, the architecture can be overfit to any dataset for a boost in performance. However, to fine-tune a model there is a computational skillset required which may be too complex for some users to make use of, a barrier that is not present with a data logger.’

***l.352 : In my copy, fig 6 has a very low quality.***

The submitted figures are of a higher quality

**** Discussion
l.377-382 : This paragraph is a bit repetitive and not really necessary in my opinion.***

I appreciate your thoughts on this paragraph, and although it is repetitive of the manuscript contents I feel it is a necessary introduction to the discussion.

***l.386 : see my comment on l. 198***

See response to comment for line 198.

***l.391-392 : I don't really agree with this comment, I think a lot can be explained of the***

***The criteria for each system to register a detection also differs performances of each when going more in detail into the data (like it is done later, L.440 for the FPR of whistle detection).***

We believe this statement is valid. It claims the rationale behind decisions cannot be observed. One can, as we do, observe the behaviour, but one cannot integrate the systems to understand the reasons for decisions, as one might for example using traditional signal processing methods.

***l.400 : "unseen acoustic data" see my comment on l.94***

See response to comment on L94.

***l.404 : The whistle detection improved the correlation coefficient but the very high rate of FP is alarming.***

**The high rate of FP is alarming, particularly at Hyskier and Shiant Isles. Upon review of the raw audio data we attribute this to elevated noise conditions above 1kHz, associated with weather. The model is not pre-exposed to the data used in this evaluation and elevated noise in frequency bins which contain whistles can cause false-positive detections. Applying site-specific fine-tuning on a subset of data will remove the false-positive rate substantially as the model weights can adapt to the range of noise levels present in the data (White *et al*., 2022). A statement about this is present in the discussion in lines 592 – 609.**

***l.416 : "when applying ..." I don't get it : was not 0.9 the threshold you chose in the first place(l.349) ?***

Our earlier corrections address this point.

***l.427 : there are other studies which did not find the same result, I think? Could it be due to this individual C-POD performing poorly? To the set-up, not favourable for this instrument? To the fact that the C-POD is performing better for NBHF species (Narrow band High frequency)? It could be interesting to include these elements to your discussion, for a more balanced (and thus more useful) conclusion.***

Our amendments to the Methods section include the line: ‘The moorings were deployed simultaneously, so represent data from three different instruments of each type.’ For this reason, we are confident that the consistency of our results determine that it is not due to an individual C-POD performing poorly. While many studies compare processing algorithms to the C-POD, particularly PAMGuard, few compare with manually labelled data, and this could be the reason our results contrast other studies. In the literature I reviewed I found our outputs to be consistent with other studies on both delphinid vocalisations and NBHF signals (e.g. Harbor Porpoise).

***l.428 : see my comment l.35 ...***

Please see my response to line 35.

***l.428 : yes. I think the sensitivity of the instruments, as well as the detection ranges, could have been introduced higher in the paper, I feel they are constitutive of the comparison.***

With respect to the general comment on this issue please see my detailed response above.

I appreciate your feedback about introducing the sensitivity of each instrument higher up in the manuscript, but I personally feel in this context they are relevant only to our discussion. We do not quantitatively compare the specific sensitivity of each instrument, nor do we have the data to compare each instrument at different sensitivity levels. All we can do is compare the two platforms (SoundTrap and C-POD) as sold out of the box with respect to detection capabilities from two processing pipelines. To introduce the sensitivity of the equipment higher up would require extensive analyses to be conducted which are frankly out of the scope of this paper. Where relevant to the interpretation of the results from our comparison the sensitivity is discussed, we do not shy away from the fact that a different recorder could change the results, but given we have three different SoundTraps and three different C-PODs in rotation throughout the experiment, I think we account for any individual instrument bias. We highlight where sensitivity may be a concern as a result of the recording environment, but I do not think we need to delve any further into the discussion of hardware.

***l.433 : ref 31, not 301***

The reference to 301 has been corrected to 31.

***l.440 : not only in April ...***

Line 440 (now line 501) has been changed to read ‘The CNN output a high rate of false-positives when accounting for both whistles and clicks, particularly during April at Hyskier and Shiant Isles (Figure 6).’

***l.440 : this paragraph is interesting, you could maybe show the signal in the suppl. material?***

Thank you for your suggestion. I have included a range of anonymised spectrograms of the anthropogenic chirp signal present in April at each of the three sites in Supplementary Figure 3. The time and frequency axis have had to be removed due to sensitivity of the data, but I agree that including the signal does provide insight to how similar these signatures are to whistles.

***l.452 : I am still lost on the threshold that was applied in the first place.***

This has been clarified earlier.

***l.461-462 : maybe "vocal" activity is not the correct term for clicks?***
This line has been edited to read: ‘We show that delphinid acoustic activity is highest between 4pm and 8am at each mooring, with higher densities of vocally active hours in the winter months (Figures 3 & 6)’.

* **Bibliography
*l.542 : could you give Editor ?***

I have amended the reference to be: M. C. Caldwell, D. K. Caldwell and P. L. Tyack, “Review of the Signature-Whistle Hypothesis for the Atlantic Bottlenose Dolphin,” In: S. Leatherwood and R. R. Reeves, Eds., The Bottlenose Dolphin, Academic Press, San Diego, 1990, pp. 199-234.

***l.604 : idem***

This reference is cited from the Proceedings Of Meetings on Acoustics which is an ASA journal based on conference proceedings, an editor was not available for citation.

***l.688 : idem***

A fuller citation has been provided: Reid, J.B., Evans, P.G.H. & Northridge, S.P. 2003. Atlas of Cetacean distribution in north-west European waters, JNCC, Peterborough.
