# Supplementary material for: Evaluating the performance of automated detection systems for long-term monitoring of delphinids in diverse marine soundscapes
Source: PLoS One. 2025 Jun 11;20(6):e0323768. doi: 10.1371/journal.pone.0323768 (PMC12157003; doi:10.1371/journal.pone.0323768)
Supplement: Attachment — Submitted filename: Response_To_Reveiwers_V2.docx [file pone.0323768.s004.docx]

Dear Editor,

We are grateful for the further comments supplied by the reviewers and thank Reviewer 2 for their support in the publication of our manuscript. Reviewer one supplied further comments, which we have responded to in detail below (1-6).

Yours Sincerely,

Dr Ellen White

School of Ocean and Earth Sciences

University of Southampton

*1. The quality of most figures in my copy is really low. Are they vector figures? If not, please convert them to vector figures for better quality.*

Thank you for raising the concern regarding figures again, however the submitted figure quality is high and is being reduced during the pdf conversion as per the review portal. The final published article will not contain the low quality as seen here.

*2. Some of the line numbers in your responses do not match the actual line numbers in your revised PDF.
For example: “Line 103, on page 5 now reads: One widely adopted ML approach makes use of Convolutional Neural Networks (CNNs) …” this sentence is in line 93. For example: “I have now removed the second ‘for which’ from line 80”, but I found the ‘for which’ in line 72.
For example: “Line 231: During post-processing a threshold can be…” is in line 223 of your revised pdf.*

I apologise for the miscommunication regarding line numbers within the feedback. I really appreciate the efforts that went into your review and have addressed each point accordingly. The line numbers will have shifted with multiple authors editing the text in response to each point and a few inconsistencies will have remained. In future I will strive to ensure this does not happen again.

*3. There are still two paragraphs in the abstract.*

The paragraphs in the abstract have been condensed into one body of text (Lines 2-18). *4. Line 101, ‘This section outlines the methodology use, beginning….’, please change ‘use’ to ‘used’.*

The use of ‘use’ is changed to ‘used’ in line 102.

*5. Adding a sentence about the overall structure of the manuscript at the end of the introduction is to give readers a clear picture of your manuscript. You can introduce each section at the beginning of that section, but please provide only a summary rather than listing details of all subsections.*

I thank the reviewer for their continued efforts at improving the manuscript. The final paragraph of the introduction does currently provide an overview of the analyses conducted within the manuscript, in order. If the reviewer has further suggestions to improve this I would welcome them, but do not see how this can be further enhanced beyond the existing text:

‘In this work we conduct the first empirical evaluation of the performances of a CNN, applied to broadband acoustic data recorded on a SoundTrap 300HF (Ocean Instruments), and the C-POD on the task of delphinid detection off the west coast of Scotland (Figure 1). An open-source multi-sound source CNN [49] is used without any network adaptation to the test data. We compare the output of both algorithms, across diverse acoustic conditions, against manually labelled recordings to evaluate their suitability in the standard processing toolkit for long-term delphinid monitoring’.

The introductory section header for the methods section (Line 102 – 105) has been condensed to:

‘This section outlines the methodology used, beginning with a detailed summary of the data acquisition process (Section 2.1), describing the analysis of acoustic data through each approach: a) manual, b) the CNN, and c) the C-POD (Section 2.2) and the evaluation metrics used in subsequent sections to assess the performance of each approach (Section 2.3).’

Given the vast detail within the results section, I feel the current introductory paragraph is needed to guide the reader through the structure of this sentence. This detail is difficult to include higher up in the introduction as the reader will require the context of the methods section to follow it. I have tweaked it (Lines 262 – 269) to read:

‘This section evaluates the performance of the C-POD and the CNN, applied to broadband acoustic data, in detecting delphinid presence across diverse underwater soundscapes. This analysis is based on 3,000 hours of acoustic data collected throughout 2019 in western Scottish waters. First, we conduct an overall comparison of click detections (Section 3.1). The output of each detector is then examined to identify temporal and seasonal patterns (Section 3.2). Sections 3.3 and 3.4 compare detector performance across varying ambient noise conditions using ROC curves and precision-recall metrics. Finally, Section 3.5 incorporates whistle detections output by the CNN to evaluate the benefits of monitoring delphinids using multiple signal types.’

*6. For figure 2, please avoid adding many texts in the caption -- a picture is worth a thousand words. If possible, please add an example of the labels used for
CNN in the figure 2, similar to the numeric labels presented for Manual Validation and C-POD.*

Regarding the caption for Figure 2, the detail provided was in line with the request of Reviewer 2 to enhance overall clarity. We feel the caption succinctly summarises the complex figure, providing clarity to the reader outside of the main text. I have edited it slightly to reduce the word count, but feel that the current text is justified against the level of detail in the figure (Lines 165 – 172):

‘***Figure 2***. Schematic depiction of the data collection and subsequent analysis workflow used for the determination of delphinid detection positive hours (dph) by a) Manual, b) CNN, and c) CPOD detection. Manual analysis applies a positive or negative label for the presence of delphinid clicks and delphinid whistles. Audio data is fed through the CNN which outputs one label per input frame (3 seconds); Ambient noise, delphinid whistle, delphinid click or vessel noise, and the total frames per class are summed per file to provide hourly presence/absence of clicks. The C-POD outputs detection positive minutes for delphinid clicks, for each minute of the recording hour, with the total sum of positive minutes converted to positive or negative detection hour.’

I thank the reviewer for their suggestion within Figure 2. In the CNN pipeline, I show the labels ‘Click/Whistle/Vessel’ as the signals which the network can detect. This is line with what is shown for Manual Validation and C-POD and so I have not added any additions to this figure. If the reviewer means to add [1,1] similar to manual validation we do not think this is appropriate as there are three label options for the CNN and to demonstrate these as binary arrays would require adding: [1,0,0], [0,1,0], [0,0,1]This is detail that we do not feel is necessary within this figure.

*7. Let’s revisit to the novelty of this manuscript. The purpose of your study is to detect the presence or absence of delphinids, and the objective of this study is to demonstrate the broader capabilities of deep learning and highlight how an off-the-shelf network—without extensive fine-tuning—can still produce meaningful insights into species presence.

I believe this work underscored the importance of the study presented in “Automated detection of marine sound sources with a convolutional neural network”, but it still lacks sufficient novelty for a new manuscript. The comparison between the performance of your CNN model and the C-POD is valuable and represents a large amount of data processing and effort. I truly appreciate your contribution to this field, but, comparing an existing CNN model (already published) with a device (C-POD) without a clear understanding of the specific detection methods used in the device is not sufficient for publication.*

While we appreciate Reviewer One's concerns regarding novelty (7), we respectfully suggest that our application of an existing neural network from published literature represents a meaningful contribution. Specifically, our work demonstrates—for the first time in this context—how deep-learning algorithms can be effectively deployed to extract species presence from acoustic recordings made in unseen marine soundscapes. This approach significantly advances accessibility for non-technical users in the field.

Our work demonstrates a rigorous comparison between established commercial data logging systems (C-POD) and emerging machine learning approaches. This comparison provides timely and valuable insights for the research community as machine learning tools are becoming standard within this domain. We provide a detailed characterisation of detection biases for both approaches, offering practitioners a nuanced understanding of each methods strengths and limitations. We expect that our analyses will enable informed decision-making about monitoring strategies based on specific research questions and environmental contexts.

The reviewers concern regarding the C-PODS proprietary algorithms we find misplaced. We note that if understanding the internal algorithms are a prerequisite for robust comparison it would invalidate a substantial body of published literature which relies on C-POD data. Our approach evaluates each system on their observable performance characteristics, which is standard practice when comparing emerging technologies. We hope that our research is accessible to researchers from a range of scientific disciplines, and we provide baseline metrics on each method of detection to educate the community on considerations they should address when designed passive acoustic monitoring projects for monitoring toothed whales.
